# *Clostridium butyricum* inhibits the inflammation in children with primary nephrotic syndrome by regulating Th17/Tregs balance via gut-kidney axis

**DOI:** 10.1186/s12866-024-03242-3

**Published:** 2024-03-23

**Authors:** Ting Li, Xiaolong Ma, Ting Wang, Wenyan Tian, Jian Liu, Wenke Shen, Yuanyuan Liu, Yiwei Li, Xiaoxu Zhang, Junbai Ma, Xiaoxia Zhang, Jinhai Ma, Hao Wang

**Affiliations:** 1 Department of Pediatrics, The First Clinical College of Ningxia Medical University, General Hospital of Ningxia Medical University, Yinchuan, China; 2 Department of Pediatrics, General Hospital of Ningxia Medical University, Yinchuan, 750004 China; 3 Department of Human Anatomy and Histology and Embryology, School of Basic Medical Sciences, Ningxia Medical University, Yinchuan, China; 4 Department of Gastroenterology, The First Clinical College of Ningxia Medical University, General Hospital of Ningxia Medical University, Yinchuan, China; 5 Department of Hepatobiliary, The First Clinical College of Ningxia Medical University, General Hospital of Ningxia Medical University, Yinchuan, China; 6 Department of Pathogenic Biology and Medical Immunology, School of Basic Medical Sciences, Ningxia Medical University, Yinchuan, China; 7 Department of Gastroenterology, General Hospital of Ningxia Medical University, Yinchuan, China; 8 College of Traditional Chinese Medicine, Ningxia Medical University, Yinchuan, China

**Keywords:** *C. butyricum*, PNS, Inflammation, Th17/Tregs, Gut microbiota, Urinary microbiota

## Abstract

**Background:**

Primary nephrotic syndrome (PNS) is a common glomerular disease in children. *Clostridium butyricum* (*C. butyricum),* a probiotic producing butyric acid, exerts effective in regulating inflammation. This study was designed to elucidate the effect of *C. butyricum* on PNS inflammation through the gut-kidney axis.

**Method:**

BALB/c mice were randomly divided into 4 groups: normal control group (CON), *C. butyricum* control group (CON+*C. butyricum*), PNS model group (PNS), and PNS with *C. butyricum* group (PNS+*C. butyricum*). The PNS model was established by a single injection of doxorubicin hydrochloride (DOX) through the tail vein. After 1 week of modeling, the mice were treated with *C. butyricum* for 6 weeks. At the end of the experiment, the mice were euthanized and associated indications were investigated.

**Results:**

Since the successful modeling of the PNS, the 24 h urine protein, blood urea nitrogen (BUN), serum creatinine (SCr), urine urea nitrogen (UUN), urine creatinine (UCr), lipopolysaccharides (LPS), pro-inflammatory interleukin (IL)-6, IL-17A were increased, the kidney pathological damage was aggravated, while a reduction of body weights of the mice and the anti-inflammatory IL-10 significantly reduced. However, these abnormalities could be dramatically reversed by *C. butyricum* treatment. The crucial Th17/Tregs axis in PNS inflammation also was proved to be effectively regulated by *C. butyricum* treatment. This probiotic intervention notably affected the expression levels of signal transducer and activator of transcription 3 (STAT3), Heme oxygenase-1 (HO-1) protein, and retinoic acid-related orphan receptor gamma t (RORγt). 16S rRNA sequencing showed that *C. butyricum* could regulate the composition of the intestinal microbial community and found *Proteobacteria* was more abundant in urine microorganisms in mice with PNS. Short-chain fatty acids (SCFAs) were measured and showed that *C. butyricum* treatment increased the contents of acetic acid, propionic acid, butyric acid in feces, acetic acid, and valeric acid in urine. Correlation analysis showed that there was a closely complicated correlation among inflammatory indicators, metabolic indicators, microbiota, and associated metabolic SCFAs in the gut-kidney axis.

**Conclusion:**

*C. butyricum* regulates Th17/Tregs balance via the gut-kidney axis to suppress the immune inflammatory response in mice with PNS, which may potentially contribute to a safe and inexpensive therapeutic agent for PNS.

**Supplementary Information:**

The online version contains supplementary material available at 10.1186/s12866-024-03242-3.

## Introduction

Primary nephrotic syndrome (PNS) characterized by massive proteinuria, hypoalbuminemia, edema, and hyperlipidemia, represents a common glomerular disease, accounting for about 90% of nephrotic syndrome in children [1, 2]. It is reported that the incidence of PNS is approximately 2-7/100,000 in children and the prevalence rate is about 16/100,000 [3]. A large amount of albumin loss in the urine leads to infection, thromboembolism, cardiovascular disease, hypovolemic crisis, anemia, and acute kidney failure [4]. At present, the treatment of PNS in children is limited to glucocorticoids and immunosuppressants. Long-term use of glucocorticoids and immunosuppressants may induce multiple adverse reactions in children, such as cushingoid symptoms, obesity, growth retardation, and bone marrow suppression [4]. Although most children respond well to steroids within four weeks, the majority of them will relapse and about half of children have a recurrence or develop steroid dependence [5]. Therefore, there is an urgent need to seek safe and effective intervention strategies for children with PNS.

PNS in children is mainly believed in associated with immune dysfunction including lymphocytes and podocytes [6, 7]. The main manifestations are the abnormal number/functions of T lymphocytes and the imbalance of the proportion of each subset [8]. A biased T helper cell 17 (Th17)/Regulatory cells (Tregs) axis in patients with PNS is thought to be closely correlated with remission and resistance [9, 10]. Tregs are reduced in children with PNS and recovered after remission [11, 12].

*Clostridium butyricum* (*C. butyricum)* is an obligate anaerobic gram-positive bacillus, its main metabolite butyric acid has been proven by many research teams including our lab to improve atherosclerosis [13], alcoholic liver disease [14], kidney ischemia-reperfusion injury [15], airway inflammation [16], and other diseases through anti-oxidative stress and reducing inflammatory response. It is worth noting that gut dysbiosis has an important impact on the proportion of butyrate-producing bacteria, and once the gut homeostasis is destroyed, it may lead to or participate in the occurrence and development of the disease [12, 17–19].

PNS is also known as idiopathic nephrotic syndrome (INS). It has been found that the proportion of butyrate-producing bacteria in the gut flora of children with INS is decreased and related to the imbalance of Th17/Tregs in peripheral blood [12]. However, the effect of *C. butyricum* intervention on PNS and its possible mechanisms are largely unclear.

This study was designed to investigate the effect of *C. butyricum* on PNS and associated underlying mechanisms via the gut-kidney axis by regulating Th17/Tregs balance, which may potentially contribute to a therapeutic agent for the control of PNS.

## Materials and methods

### Experimental animals and PNS model

All experiments were performed according to Animal Use Guidelines and approved by the Ethics Committee of Ningxia Medical University (No.2022-206). Forty-eight male 6-week-old BALB/c mice (22±1g) were obtained from Beijing Huafukang Bio-Technology. Co., LTD (Beijing, China). Mice were fed in the Laboratory Animal Research Center of Ningxia Medical University (Yinchuan, China) under a 12 h light and dark cycle with free access to food and water. The mouse model of PNS was established by a single injection of doxorubicin hydrochloride (DOX; MCE, USA) 10mg/kg through the tail vein [20]. One week later, the mice were placed in a mice metabolic cage to collect 24 h urine and measure 24 h urine protein. After successful modeling, the subsequent experiments were performed.

### Bacterial preparation

*C. butyricum* was a vacuum freeze-dried strain provided by the China General Microbiological Culture Collection Center (CGMCC, strain number 1.5205). PYG MEDIUM (modified; Shandong, China) was used to resuscitate the bacteria. The bacteria were cultured in an anaerobic incubator (5% carbon dioxide) at 37 °C for about 24 h, and the fully grown bacteria were visible. The colonies were placed in 10% skim milk to make a freeze-dried powder and stored at -80 °C. During the intervention, PYG MEDIUM was used daily to resuscitate *C. butyricum* lyophilized powder in an anaerobic incubator (5% carbon dioxide) at 37 °C for 24 h, centrifuged at 3000×g for 5 min, and resuspended in sterile saline. The experimental final concentration was 1×108 CFU/mL.

### Experimental design

**Fig. 1:**
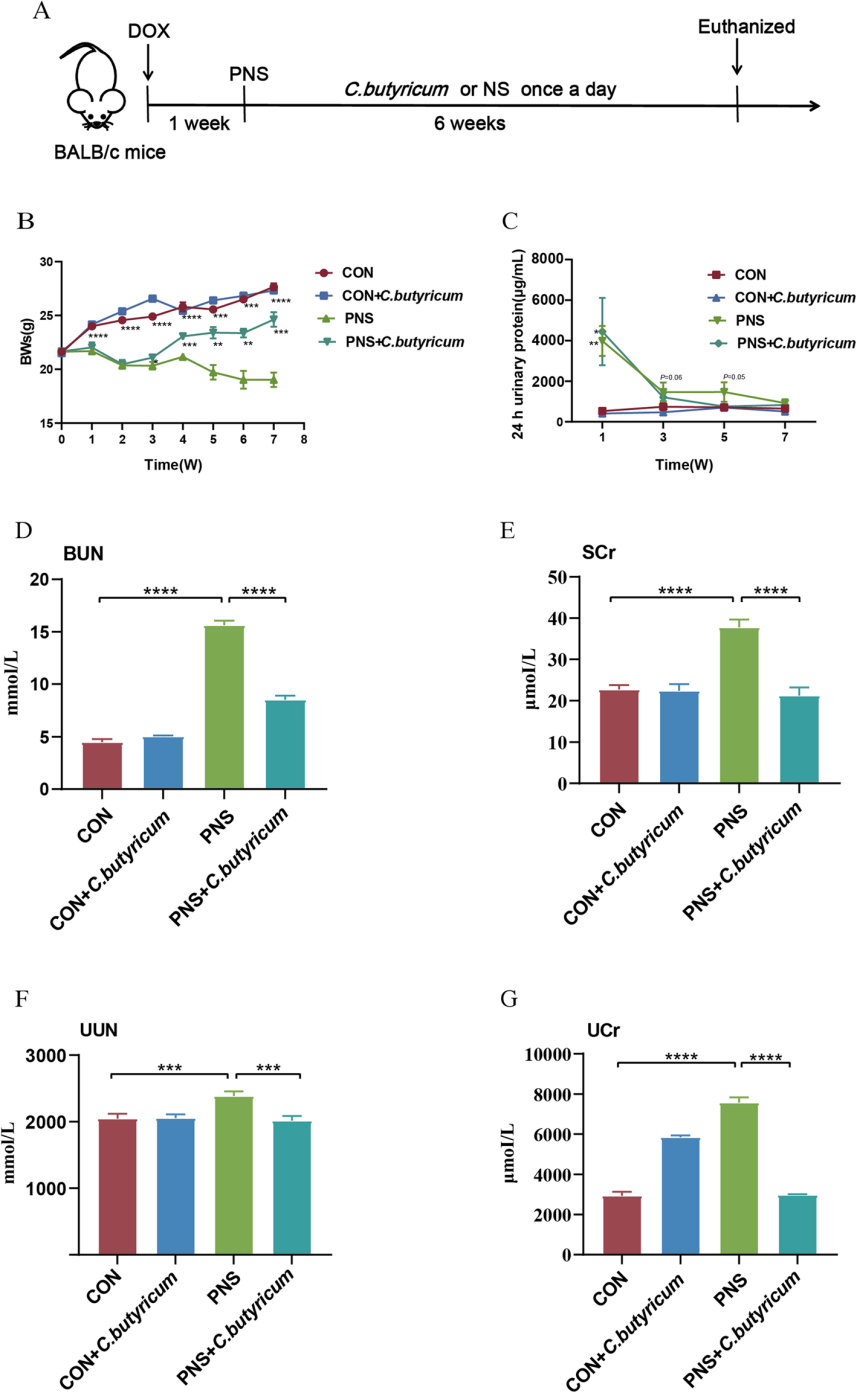
The impacts of *C. butyricum* treatment on BWs, 24 h urinary protein and kidney function in DOX-induced PNS mice. Experimental design time diagram (**A**). BWs: Body weights (**B**). 24 h urine protein of mice in diverse groups (**C**). BUN: Blood urea nitrogen (**D**). SCr: Serum creatinine (**E**). UUN: Urine urea nitrogen (**F**). UCr: Urine creatinine (**G**). Data were expressed as mean±SD. *P < 0.05, **P < 0.01, *** *P* < 0.001, ****P < 0.0001. All experiments were performed in triplicate

The time diagram of the experimental design is shown in Fig. 1A. The mice were randomly divided into 4 groups (12 mice/group): CON, CON+*C. butyricum,* PNS, and PNS+*C. butyricum*. The CON+*C. butyricum* and PNS+*C. butyricum* groups were given 200μL *C. butyricum* by gavage once a day for 6 weeks. Meanwhile, mice in CON and PNS groups were treated with 200 μL normal saline at the same time. The 24 h urine was collected every 2 weeks using a mice metabolic cage, and the 24 h urine protein was measured by Bradford protein detection kit (Thermo Fisher, USA) to observe the changes of urine protein in mice. During the experiment, the body weights (BWs) of the mice were monitored weekly. After 6 weeks of gavage, fresh stool, and urine samples were collected and immediately frozen at -80 °C for subsequent analysis. At the termination of the experiment, mice were anesthetized with isoflurane inhalation (0.41 mL/min at 4 L/min fresh gas flow, application concentration 2%) was performed under the IACUC protocol and euthanized via transcardiac perfusion. The blood samples were taken from the orbit of mice and collected in tubes containing ethylenediaminetetraacetic acid (EDTA), and centrifuged at 4 °C (600×g for 10 min) to obtain plasma samples, which were stored at -80 °C for further study.

### Plasma and urine biochemistry tests

Blood and urine samples were measured for the following biochemical properties: Blood urea nitrogen (BUN), serum creatinine (SCr), urine urea nitrogen (UUN), and urine creatinine (UCr). BUN and UUN were detected by urease method with a urea nitrogen test box; SCr and UCr were measured by creatinine assay kit using the sarcosine oxidase method (Nanjing Jiancheng Bioengineering Institute, Nanjing, China).

### Kidney tissue pathological section staining

After the sacrifice of the mice, the kidney tissues were immediately fixed in 4% paraformaldehyde, washed with tap water, and dehydrated with ethanol, paraffin-embedded sections (5μm) were placed on slides. Hematoxylin & eosin (HE) staining (Servicebio, NO.G1004, NO.G10001, Wuhan China), Masson staining (Solarbio, NO.G1340, Beijing, China), Periodic Acid Schiff (PAS) staining (Solarbio, NO.G1281, Beijing, China), and Periodic acid-silver methenamine (PASM) staining (Solarbio, NO.G1790, Beijing, China) were performed on the sections according to the standard protocols.

After completing the above staining steps, the sections were observed and photographed under a microscope (Aomori Olympus glass slide, Japan) at 40X. The kidney interstitial cell infiltration score was evaluated according to HE staining, semi-quantitative assessment of the degree of inflammatory cell infiltration by counting the percentage of inflammatory cell infiltration area and classified as follows: 0 (nil), 1 (<25%), 2 (25–50%), 3 (50–75%), and 4 (>75% of tubulointerstitial fields). Each score was determined by evaluating three sections per kidney and five non-overlapping visual fields per section [21]. Image J software (National Institutes of Health Bethesda, MD) was used to quantify the percentage of PAS-stained glycogen-covered area in the total image area and the percentage of kidney interstitial fibrosis area in the total image area. Observers were blinded to the experimental groups.

### Determination of lipopolysaccharides (LPS) levels in kidney and gut

The Limulus amebocyte lysate kit (Xiamen Bioendo Technology Co., Ltd., Xiamen, China) was used to detect LPS levels in the intestine and kidneys of mice according to the manufacturer's instructions. Initially, the endotoxin standard solution was prepared according to the instructions. 100 μL of diluted intestine/kidney tissue homogenate supernatants (1:30/1:20 dilution with endotoxin-free water), endotoxin standard solution, and bacterial endotoxin test water were added into endotoxin-free test tube respectively. Bacterial endotoxin test water was used as a negative control, and endotoxin standard solution was used as a positive control. At the initial time point, 100μL of the Limulus amebocyte lysate agent was added to each tube and incubated at 37°C for 7 min. Then, 100 μL of chromogenic substrate was added to each tube and incubation at 37°C for 6 min. After the incubation, 500 μL of azo reagent 1, azo reagent 2, and azo reagent 3 were added in turn and allowed to stand for 5 min. The optical density was measured at 545 nm using a microplate reader (Thermo Scientific, Waltham, USA).

### Enzyme-linked immunosorbent assay (ELISA)

Kidney tissue homogenate supernatants were collected to determine the concentration of cytokines. Interleukin (IL)-6, IL-10, and IL-17A in the kidney were determined by using an ELISA kit (Proteintech, Wuhan, China) according to the manufacturer's instructions. The optical density at a wavelength of 450 nm was measured with an automatic microplate reader (Thermo Fisher Science Inc., USA).

### Quantitative real-time PCR

According to the manufacturer's protocol, total RNA was extracted from kidney tissue using the RNA Extraction kit (Omega, USA), and the UEIris RT mix with DNase (All-in-One) (UE, China) was used to synthesize cDNA. Then, RT-qPCR was performed using Universal SYBR Green qPCR Supermix (UE, China). The expression of the target gene was normalized by GAPDH. All experiments were carried out in three independent experiments. Primer sequences (Sangon Biotech, Shanghai, China) are shown in Table 1.

**Table 1:** List of primers used for qRT-PCR

Gene	Forward Primer (5′→3′)	Reverse Primer (5′→3′)
Keap1	GCTCAACCGCTTGCTGTATGC	CATCCGCCACTCATTCCTCTCTG
Nrf2	AGCACAGCCAGCACATTCTCC	GACCAGGACTCACGGGAACTTC
HO-1	AAGACCGCCTTCCTGCTCAAC	TCTGACGAAGTGACGCCATCTG
JAK2	AGTGTGGAGATGTGCCGCTATG	AGGTGCTCTTCAGTGCTGTGC
STAT3	CGATGCCTGTGGGAAGAGTCTC	TGTCACTACGGCGGCTGTTG
RORγt	TCTACACGGCCCTGGTTCT	ATGTTCCACTCTCCTCTTCTCTG
GAPDH	GGTTGTCTCCTGCGACTTCA	TGGTCCAGGGTTTCTTACTCC

### Western blot

The kidney tissue protein was extracted using the whole protein extraction kit (Keygen, NO.KGP250, China) according to the manufacturer's protocol. Protein concentrations were detected with a BCA protein assay kit (Keygen, No.KGP902, China). 30 μg protein was added to each well of SDS-polyacrylamide gel (SDS-PAGE) for electrophoresis. The voltage of the concentrated gel was 90 V, and the voltage was changed to 120 V when the protein was transferred to the separation gel. After electrophoresis, the protein was transferred to the methanol-soaked polyvinylidene fluoride (PVDF) membrane (Millipore, Bedford, USA). Cell membranes were then blocked with 5% skim milk at room temperature for 2 h and incubated overnight at 4 ℃ with primary antibody including Heme oxygenase-1 (HO-1) rabbit mAb (1:5000 dilution, ABclonal, NO.A19062, China), signal transducer and activator of transcription 3 (STAT3) rabbit mAb (1:1000 dilution, ABclonal, NO.A19566, China), purified anti-retinoic acid-related orphan receptor gamma t (RORγt) Antibody (1:500, BioLegend, NO.603152, USA), Janus kinase 2 (JAK2) rabbit mAb (1:1000 dilution, CST, NO.3230S, USA), and mouse monoclonal GAPDH (1:5000 dilution, Proteintech, NO.60004-1-Ig, China). After washing with TBST buffer for 3 times, they were incubated with HRP-conjugated Affinipure Goat Anti-Rabbit IgG (H+L) (1:5000 dilution, Proteintech, NO.SA00001-2, China), HRP labeled Goat Anti-Mouse IgG (1:5000 dilution, Abbkine, NO.A21010, China), or HRP-conjugated Affinipure Goat Anti-Rat IgG (H+L) (1:5000 dilution, Proteintech, NO.SA00001-15, China) at room temperature for 1 h. After washing membranes with TBST buffer for 3 times, ECL chemiluminescence kit (Keygen NO.KGP1127, China) and SH-Compact 523 (Shenhua Technology Co., Ltd., China) were used for imaging and detection. Image J software (National Institutes of Health, Bethesda,USA) was used to analyze the gray value of the image.

### Flow cytometry analysis

The cell suspension of the spleen was prepared. The spleen was ground first, centrifuged at 400×g, 4 ℃ for 5 min after passing through a 300-mesh filter membrane, and the supernatant was discarded. An appropriate amount of red blood cell lysate was added. After standing on ice for 5 min, the sample was centrifuged at 400×g for 5 min, the supernatant was discarded, and analyzed again according to the situation. Then RPMI 1640 medium was used to wash the cells. Finally, the cell concentration was adjusted to 1×106 cells/mL for subsequent detection.

Peripheral blood mononuclear cell (PBMC) suspension was prepared as described below. The peripheral blood of mice was collected in an anticoagulant tube containing EDTA, centrifuged at 600×g, 4 ℃ for 10 min and the plasma was frozen at -80 ℃. The remaining blood cell-containing liquid was transferred to the centrifuge tube and mixed with an appropriate amount of erythrocyte lysis solution. After standing on ice for 10 min, centrifuged at 4 ℃, 400×g for 5 min, discarded the supernatant, and lysed again according to the situation. The cells were washed with RPMI 1640 medium. Finally, the cell concentration was adjusted to 1×106 cells/mL for subsequent measurement.

The colon cell suspension was prepared. After the colon was removed, the colon was washed with normal saline, the adipose tissue and feces were removed, and the longitudinal dissection was performed. The intestinal segment was cut into about 1 cm, added with RPMI 1640 medium, and placed in a shaker at 37℃ for 30 min (shaken every 10 min for 10 s). After shaking for 15 s, the intestinal segment was allowed to sink for a moment to collect all the liquid, passing through a 200-mesh filter membrane (2 times) and collecting into a centrifuge tube. At the same time, a nylon wool column was prepared: glass wool was loosely filled in a 10 mL syringe and the column was firstly infiltrated with RPMI 1640 medium, the filtrate was continuously filtered 3 times. Then the collected intestinal cell fluid was filtered, and the column was washed with RPMI 1640 medium. All the liquid was collected and centrifuged for 500×g, 4 ℃ for 5 min, and the supernatant was discarded. The RPMI 1640 medium was added and centrifuged for 600×g, 4 ℃ for 20 min to collect the white membrane in the middle part. The RPMI 1640 medium was added and centrifuged for 400×g, 4 ℃ for 5 min. The supernatant was discarded and an appropriate amount of RPMI 1640 was added to resuspend the cells. The cell concentration was adjusted to 1×106 cells/mL for subsequent detection.

Flow cytometry was used to detect the percentages of Treg and Th17 cells in diverse tissues. For Treg cell staining, CD4-FITC (eBioscience, NO.2344796, USA) was used for surface labeling and eBioscience™ Foxp3/Transcription Factor Staining Buffer Set (Thermo Fisher, USA) was elicited to fix and penetrate the cells. After that, the transcription factor forkhead box p3 (Foxp3)-PE (eBioscience, NO.4307350, USA) was added for intracellular labeling, and incubated at 4 °C for 30 min in the dark. For Th17 cell staining, cells were stimulated at 37 °C for 1 h using a 500× cell stimulation cocktail (Thermo Fisher, USA), followed by cell staining, including CD4-FITC (eBioscience, NO.2344796, USA) and IL-17A-PE (eBioscience, NO.4306419, USA), incubated at 4 °C in the dark for 30 min. Finally, prepared samples were measured and analyzed by Beckman Cyto FLEX flow cytometer (Beckman Bioscience, USA).

### Gut and urethra microbiota analysis

After 6 weeks of intervention with *C. butyricum*, 6 mice in each group were randomly selected to collect fresh feces in sterile cages and 3 mice in each group were randomly selected to collect fresh urine in sterile metabolic cages. The collected feces and urine were immediately stored at -80 ℃ until DNA was extracted.

The total DNA was extracted by Omega Mag-bind soil DNA kit (Omega M5636-02) and the DNA was quantified by Nanodrop, the quality of DNA extraction was detected by 1.2% agarose gel electrophoresis. The DNA in the sample was extracted using ultra-clean kits and reagents. UsingO5® High-Fidelity DNA Polymerase of TransGen Biotech (Beijing, China) with the primers 338F-5′ ACTCCTACGGGAGGCAGCAG 3′, 806R-5′ GGACTACHVGGGTWTCTAAT 3′ to amplified the hypervariable region V3-V4 of 16S rRNA sequence in bacterial DNA samples by PCR amplification instrument (ABI 2720, USA). A negative control during the PCR amplification of the target fragment. The negative control can detect microbial contamination such as environment and reagents, and any sample group with negative control amplification bands cannot be used for subsequent experiments. Quant-iT PicoGreen dsDNA Assay Kit fluorescence reagent and Microplate reader (BioTek, FLx800) quantitative instrument were used for fluorescence quantification of PCR amplification recovery products. The sequencing library was prepared using Illumina's TruSeq Nano DNA LT Library Prep Kit, and the library was subjected to final fragment selection and purification by 2% agarose gel electrophoresis. Finally, the sequencing was performed on a MiSeq sequencer by Suzhou Panomix Biomedical Technology Co., Ltd., China.

For the 16S rRNA gene of bacteria, Greengenes was selected as the reference database (Release 13.8). Using QIIME2 (2019.4) analysis software, the unmatched primer sequences were discarded first, and then the DADA2 plug-in was used for data processing such as quality filtering, denoising, splicing, and chimera removal. The obtained sequences were merged according to 100% sequence similarity to generate characteristic sequence ASVs and abundance data tables. Using the method of rarefaction, a certain number of sequences were randomly selected from each sample to reach a unified depth to predict the ASVs and their relative abundance that can be observed in each sample at the sequencing depth, which was used for subsequent species composition analysis, Alpha diversity analysis, Beta diversity analysis, and species difference analysis. Observed species, Chao1, and Shannon indices were used as measurements of alpha-diversity of the microbial communities. Jaccard-based principal coordinate analysis (PCoA) and nonmetric Multidimensional scaling (NMDS) analyses revealed beta diversity. For comparing the differences in species composition between samples, the Wayne diagram was used for community analysis, the heatmap was used for species composition analysis, and the PCA was used to analyze the differences in species composition between samples. LEfSe analysis was used to search for marker species between groups.

### Measurement of the feces and urine SCFAs concentrations

Ether was obtained from Titan (Shanghai, China). Phosphoric acid was obtained from Sinopharm (Shanghai, China). Acetic acid, propionic acid, isobutyric acid, butyric acid, isovaleric acid, valeric acid, 4-methylvaleric acid, and caproic acid were all obtained from Sigma-Aldrich (Shanghai, China). The 100 mg/mL stock solutions of 6 SCFAs (acetic acid, propionic acid, isobutyric acid, butyric acid, isovaleric acid, and valeric acid) and caproic acid were prepared by water and ether methods, respectively, and a series of working standard solutions were obtained by dilution.

Fecal standards were prepared, and metabolites were extracted. The internal standard (4-methylvaleric acid) was prepared with ether to 375 μg/mL, 200 μL series of working standard solutions of 6 acids, 100 μL 15 % phosphoric acid, 20 μL series of working standard solutions of hexanoic acid, 20 μL internal standard, and 260 μL either were mixed to prepare ten standard curve points, covering 0.02 to 500 μg/mL (0.02, 0.1, 0.5, 2, 10, 25, 50, 100, 250, 500 μg/mL). A total of 50 mg fecal samples were homogenized with 100 μL of 15% phosphoric acid, 20 μL of 375 μg/mL internal standard (4-methylpentanoic acid) solution, and 280 μL ether for 1 min, centrifuged at 4 °C 12000 rpm for 10 min. The supernatant was analyzed by gas chromatograph-mass spectrometric (GC-MS).

Urine standards were prepared, and metabolites were extracted. The internal standard (4-methylvaleric acid) was prepared with ether to 75 μg/mL. A series of working standard solutions of 200 μL six acids, 100 μL 15% phosphoric acid, 20 μL caproic acid, 20 μL internal standard, and 260 μL ether were mixed to prepare ten standard working solutions, covering from 0.02 to 100 μg/mL (0.02, 0.1, 0.5, 1, 2, 5, 10, 25, 50, 100 μg/mL). An appropriate amount of sample was taken in a 2 mL centrifuge tube, added with 50 μL 15% phosphoric acid, and then added with 10 μL of 75 μg/mL internal standard (isocaproic acid) solution and 140 μL of ether for homogenization for 1 min, centrifuged at 4 °C 12000 rpm for 10 min, and the supernatant was analyzed by GC-MS.

The GC analysis was performed on trace 1310 gas chromatograph. The chromatographic column was Agilent HP-INNOWAX capillary column (30 m×0.25 mm ID×0.25 μm) (Thermo Fisher Scientific, USA). MS detection of metabolites was performed on ISQ LT (Thermo Fisher Scientific, USA).

### Statistical analysis

GraphPad Prism version 9.0 (GraphPad Software Inc., La Jolla, CA, USA) and the statistical package for the social sciences (SPSS) 23.0 software (IBM Inc., Armonk, NY, USA) were used for statistical analyses. All experimental data were expressed as the mean±SD of at least three independent experiments. One-way analysis of variance was used to compare the mean values of variables among groups. After that, Tukey's post hoc test was used to determine the significance of pairwise comparison of mean values between groups. In addition, the expression correlation was analyzed by Spearman correlation coefficient assay. *P* < 0.05 was considered statistically significant.

## Results

### *C. butyricum* treatment improved BWs, 24 h urinary protein and kidney insufficiency in mice with PNS

At the beginning of the experiment, there was no significant difference in BWs among the 4 groups of mice. After successful modeling, the mice in the model group were given *C. butyricum* or normal saline for 6 weeks. It was found that compared to the CON group, the BWs of the mice in the PNS group decreased significantly in the experiment (*P* < 0.0001), while the weights in the PNS+*C. butyricum* groups were increased gradually after 2 weeks of dietary *C. butyricum* supplement (*P* < 0.01) (Fig. 1B). 24 h Urinary Protein was significantly increased (*P*<0.01) at 1 week after DOX injection, whereas *C. butyricum* treatment significantly reduced about 70% of urine protein after 3 weeks and 80% of urine protein at 5 weeks (Fig. 1C).

Urea nitrogen and creatinine in plasma and urine were measured to evaluate kidney function. Compared to the CON group, the levels of BUN and SCr in the PNS group were significantly increased (*P* < 0.0001), while the levels of BUN and SCr in the PNS+*C. butyricum* group were significantly decreased (*P* < 0.0001) (Fig. 1D and E). Similarly, the levels of UUN (*P* < 0.001) and UCr (*P* < 0.0001) in the urine of the PNS group showed an increase in comparison with the CON group, and the levels of UUN (*P* < 0.001) and UCr (*P* < 0.0001) in the PNS+*C. butyricum* groups were attenuated significantly (Fig. 1F and G). Collectively, *C. butyricum* could ameliorate the functional impairment of the kidney in mice with PNS.

*C. butyricum* treatment alleviated the pathological progress of PNS mice.

**Fig. 2:**
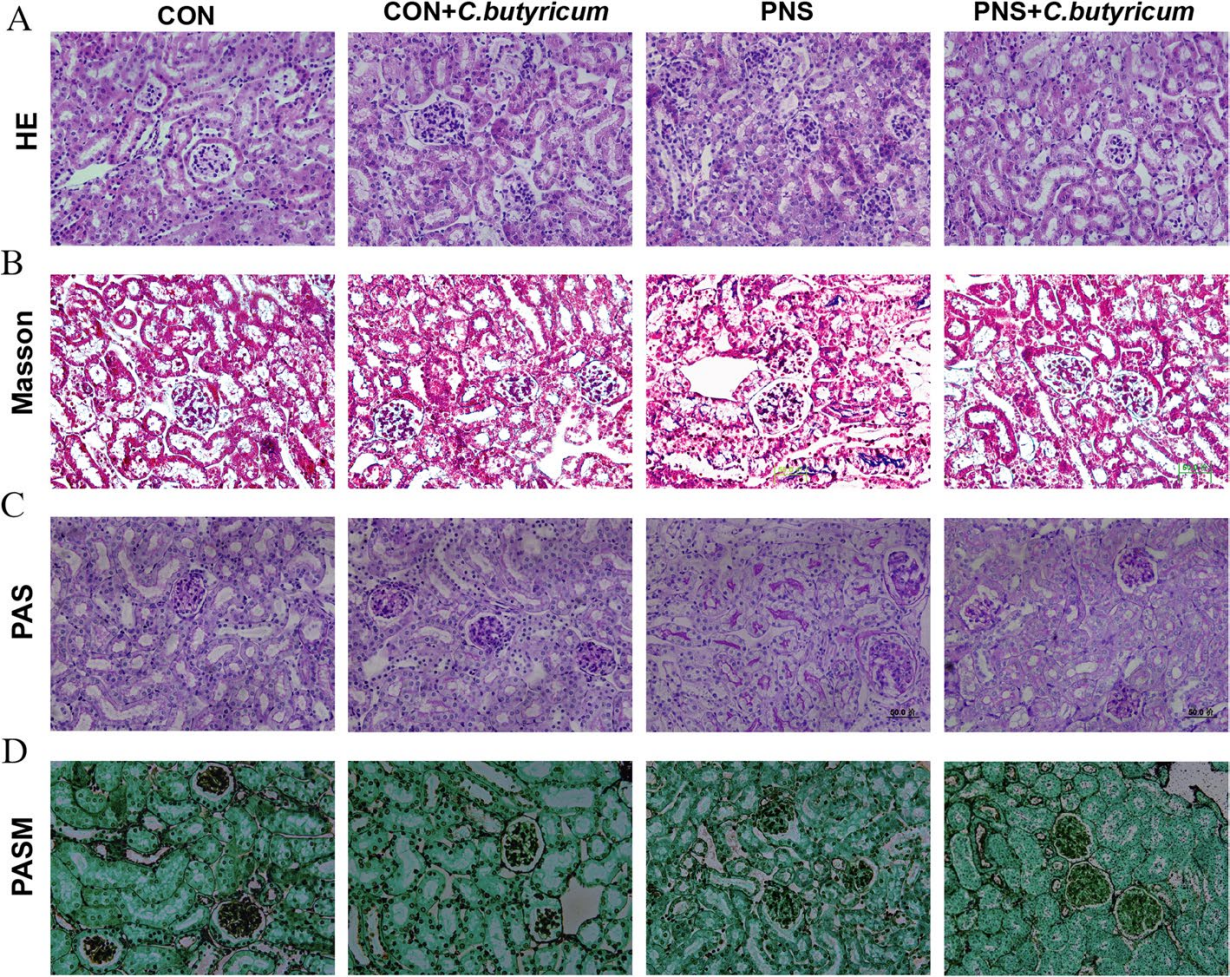
The effect of *C. butyricum* treatment on pathological progress of PNS mice. Representative images of kidney pathological staining including HE: hematoxylin and eosin (**A**), Masson's trichrome stain (**B**), PAS: periodic acid Schiff (**C**), PASM: periodic acid-silver methenamine (**D**)

To confirm whether *C. butyricum* treatment could effectively affect the pathological changes of PNS, kidney sections were stained with HE, PAS, Masson, and PASM. As expected, compared to the CON group, HE staining of the kidney in the PNS group showed increased tubular epithelial cell volume, irregular lumen, interstitial edema, and inflammatory cell infiltration (Fig. 2A, Fig .1A), and Masson staining showed increased kidney interstitial fibrosis (Fig. 2B, Fig .1B), PAS staining displayed glycogen deposition in the glomeruli and tubules (Fig. 2C, Fig .1C). In addition, we also observed partial disruption of glomerular basement membrane in the PNS group by PASM staining, whereas these changes were improved by dietary *C. butyricum* administration (Fig. 2D). Taken together, *C. butyricum* treatment possessed the capacity for attenuating the pathological lesions of PNS in mice.

*C. butyricum* treatment reduced endotoxin levels in the gut-kidney axis of PNS mice.

**Fig. 3:**
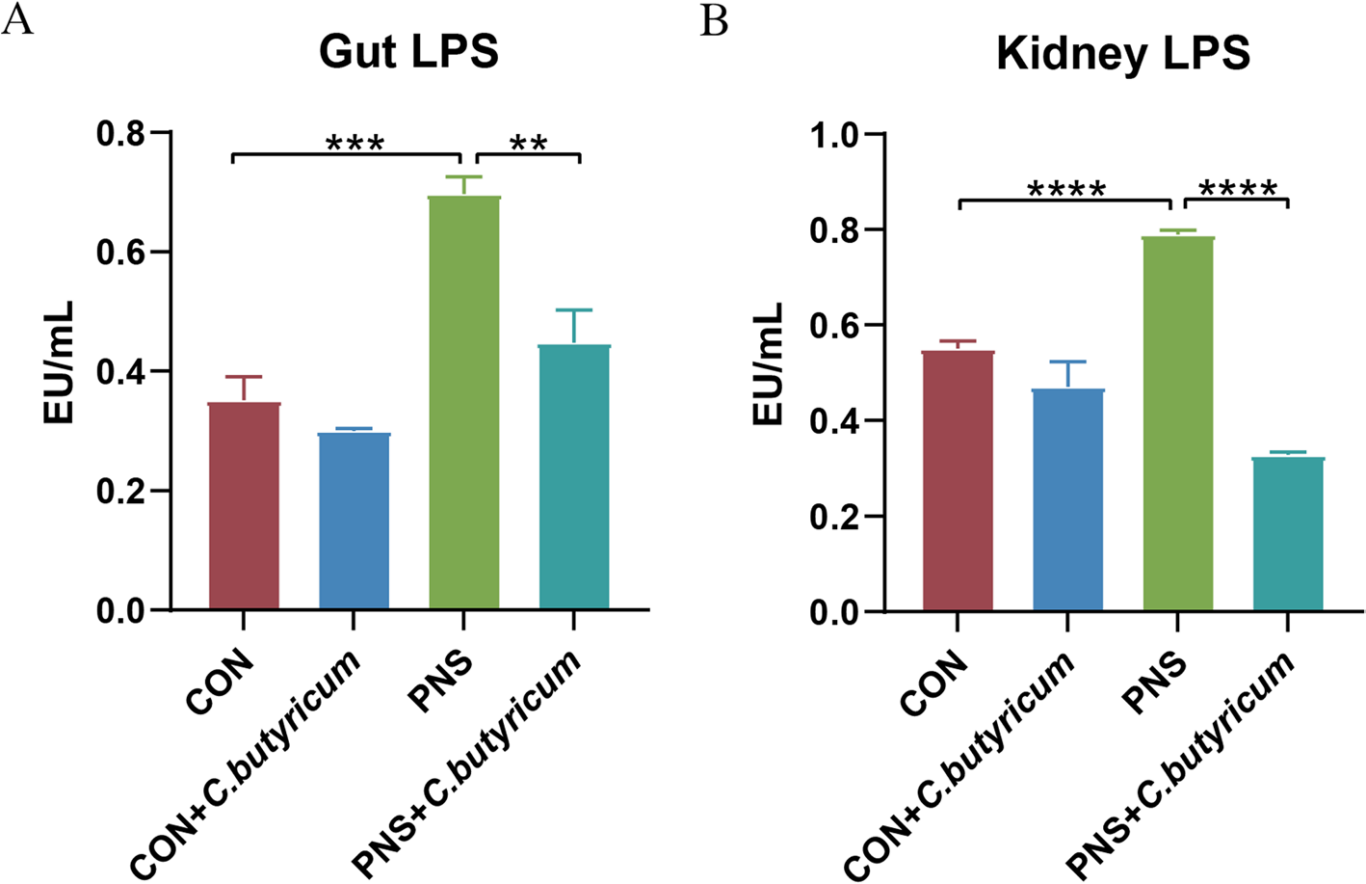
*C. butyricum* treatment reduced lipopolysaccharide (LPS) level in mice with PNS. Gut LPS levels (**A**). Kidney LPS levels .(**B**) Data were expressed as mean±SD. **P < 0.01, ***P < 0.001, ****P < 0.0001. All experiments were performed in triplicate

LPS derived from gut pathogenic bacteria plays a vital role in the promotion of inflammation. Thus, the level of LPS in the gut-kidney axis was measured and found that compared with the CON group, LPS in the gut and kidney of the PNS mice was significantly increased (*P* < 0.001, *P* < 0.0001), but markedly decreased after *C. butyricum* treatment (*P* < 0.01, *P* < 0.0001), indicating that *C. butyricum* could weaken LPS generation and translocation from gut to kidney (Fig. 3A and B).

*C. butyricum* suppressed the inflammation by regulating the balance of Th17/Tregs in mice with PNS.

**Fig. 4:**
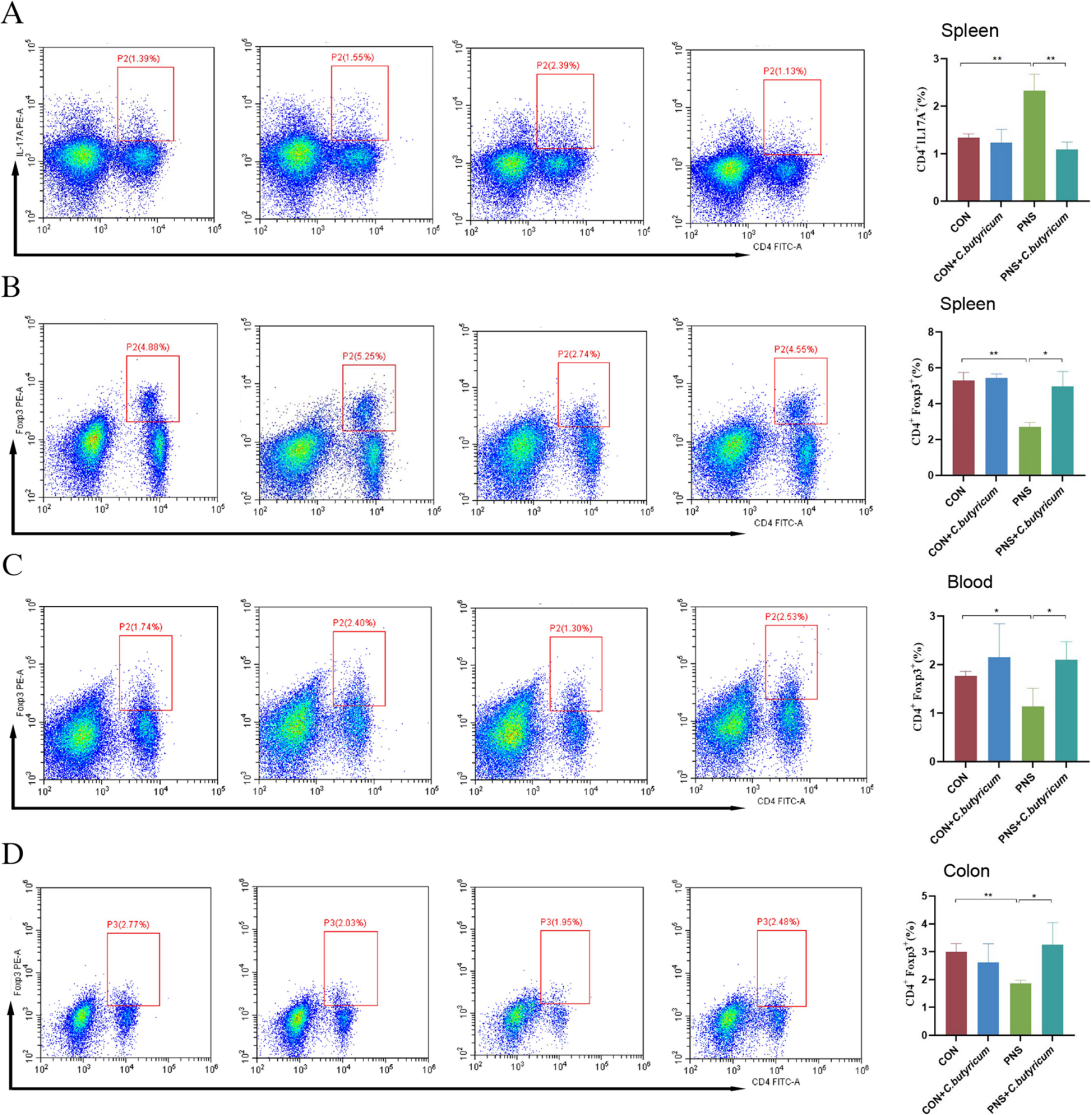
Regulation of Th17/Tregs balance by dietary *C. butyricum* treatment in mice with PNS. Flow cytometry analysis was used to separately determine the proportions of splenic Th17 cells (**A**), splenic Treg cells (**B**), peripheral blood Treg cells (**C**) and colon Treg cells (**D**) in diverse groups. Data were expressed as mean±SD. *P < 0.05, **P < 0.01. All experiments were performed in triplicate

Clinical studies have demonstrated that there is an imbalance of Th17/Tregs in the peripheral blood of PNS children [12]. In order to further evaluate the effect of *C. butyricum* on Th17/Tregs balance in PNS, we used flow cytometry to detect Treg cells and Th17 cells in different tissues. Compared with the CON group, the expression of IL-17A in the spleen of PNS was significantly increased (*P* < 0.01), while the expression of IL-17A was significantly decreased after *C. butyricum* treatment (*P* < 0.01) (Fig. 4A). Meanwhile, we found that compared to the CON group, the expression of Foxp3 in the spleen, peripheral blood, and colon of the PNS group was significantly decreased (*P* < 0.01, *P* < 0.05, *P* < 0.01), while which was conversed by dietary *C. butyricum* administration (*P* < 0.05) (Fig. 4B to D). The above suggested that probiotic *C. butyricum* could effectively attenuate the impeded Th17/Tregs balance in mice with PNS.

**Fig. 5:**
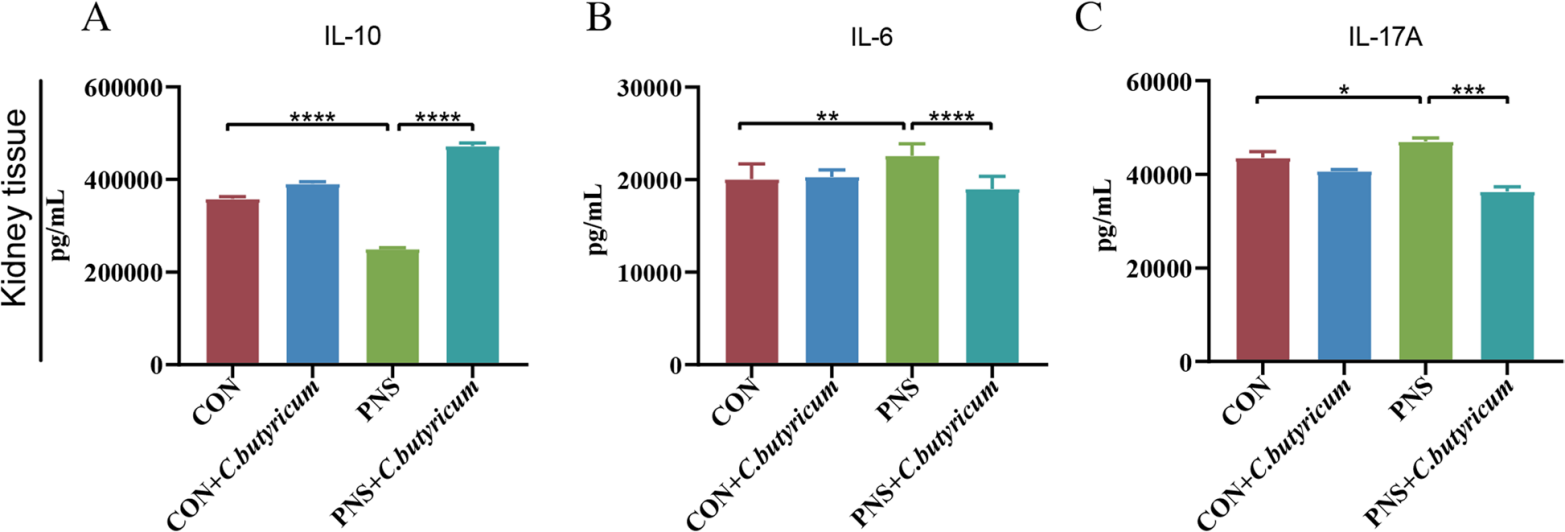
Suppression of the immune inflammatory reaction by dietary *C. butyricum* treatment in mice with PNS. Kidney tissue was elicited to determine the concentrations of IL-10 (**A**), IL-6 (**B**) and IL-17A (**C**). Data were expressed as mean±SD. *P < 0.05, **P < 0.01, ***P < 0.001, ****P < 0.0001. All experiments were performed in triplicate

Next, we identified the anti-inflammatory effect of *C. butyricum* on PNS. The results showed that the level of anti-inflammatory IL-10 in the kidney of the PNS model was significantly decreased (*P* < 0.0001) (Fig. 5A) and the levels of pro-inflammatory IL-6 and IL-17A in the kidney were increased (*P* < 0.01, *P* < 0.05) (Fig. 5B and C). After the treatment with *C. butyricum*, the kidney levels of IL-10 (*P* < 0.0001), IL-6 (*P* < 0.0001), and IL-17A (*P* < 0.001) were significantly ameliorated (Fig. 5A to C). In summary, *C. butyricum* could suppress the inflammation in mice with PNS.

*C. butyricum* treatment alleviated the inflammation through HO-1/STAT3/RORγt signaling pathway in mice with PNS.

**Fig. 6:**
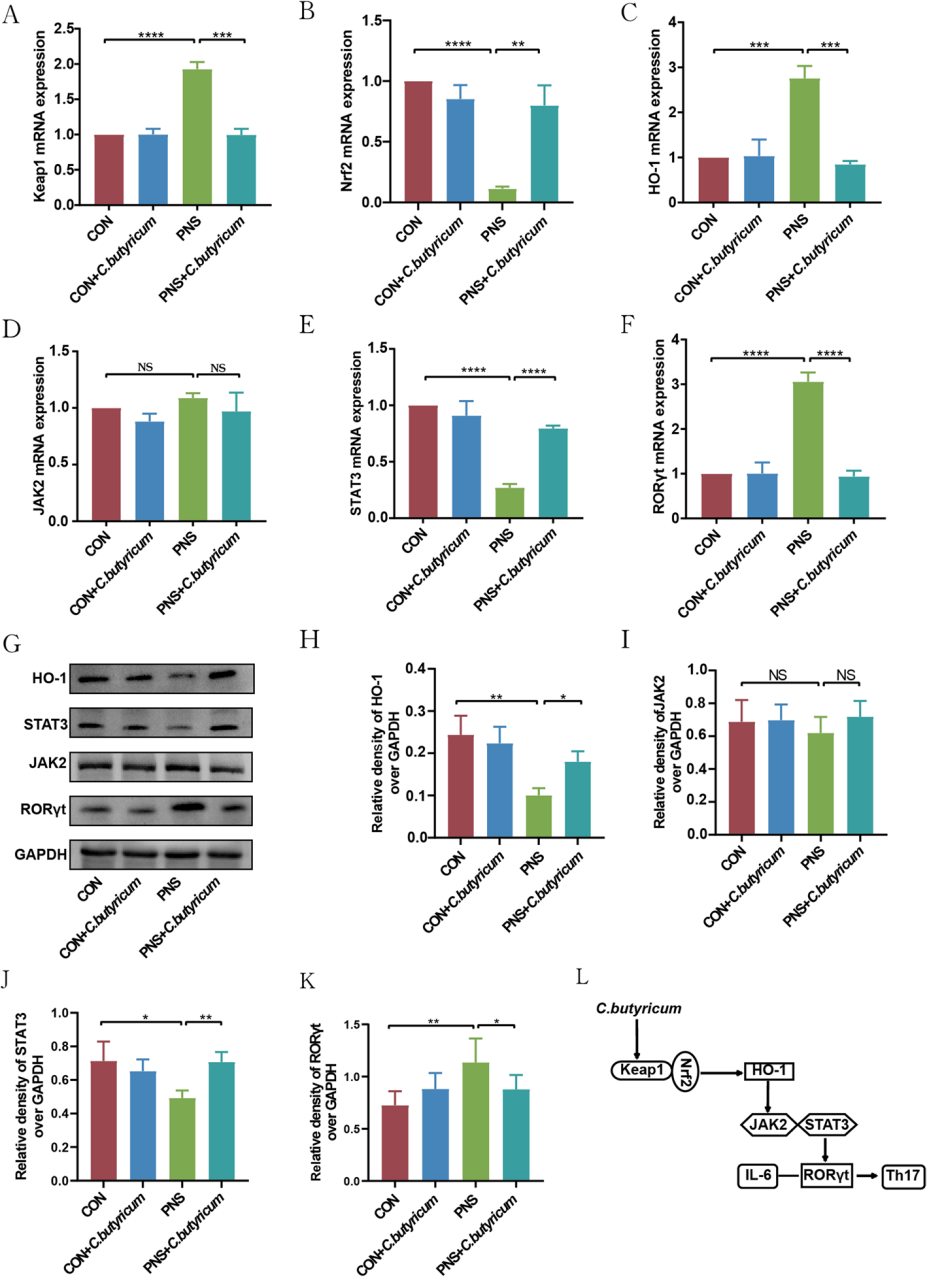
*C. butyricum* alleviated the immune inflammatory response in PNS through the HO-1/STAT3/RORγt signaling pathway. The mRNA levels of Keap1 (**A**), Nrf2 (**B**), HO-1 (**C**), JAK2 (**D**), STAT3 (**E**), and RORγt (**F**) in the kidney tissues. Representative western blot images and statistical results of HO-1 (**G**,**H**), JAK2 (**G**,**I**), STAT3 (**G**,**J**), and RORγt (**G**,**K**) expressions of protein levels in the kidney tissues. HO-1/STAT3/RORγt pathway diagram (**L**). Data were expressed as mean±SD. *P < 0.05, **P < 0.01, ***P < 0.001, ****P < 0.0001. All experiments were performed in triplicate

To elucidate the anti-inflammatory mechanism of *C. butyricum,* we detected the transcriptional expression levels of Kelch-like ECH-associated protein 1 (Keap1), nuclear factor erythroid2-related factor 2 (Nrf2), HO-1, STAT3, and JAK2 in kidney tissues. The results showed that compared with the CON group, the mRNA expression levels of Keap1, HO-1, and RORγt in the PNS were significantly increased (all *P* < 0.0001) (Fig. 6A, C, and F), while the mRNA expression levels of Nrf2 and STAT3 were significantly decreased (all *P* < 0.0001) (Fig. 6B and E). However, *C. butyricum* treatment significantly alleviated the mRNA expression levels of Keap1, HO-1, and RORγt (all* P* < 0.001), as well as elevated the mRNA expression levels of Nrf2 and STAT3 (*P* < 0.01, *P* < 0.0001). In addition, the expression of JAK2 showed no significant change in mice of diverse groups (Fig. 6D).

The protein expressions of HO-1, JAK2, STAT3, and RORγt were further measured by western blot. The results showed that compared with the CON group, the expression levels of HO-1 (*P* < 0.01) (Fig. 6G and H) and STAT3 (*P* < 0.05) (Fig. 6G and J) in the kidney tissue of the PNS group were notably decreased, which could be rectified by *C. butyricum* treatment. In addition, abnormal elevated RORγt in PNS model was completely restored by *C. butyricum* persistent intervention (*P* < 0.05) (Fig. 6G and K). The expression of JAK2 was not significantly altered among diverse groups (*P* > 0.05) (Fig. 6G and I), indicating that *C. butyricum* predominantly regulated STAT3 rather than JAK2.

It is concluded that *C. butyricum* may lead to the instability of Keap1-Nrf2 binding by inhibiting the expression of Keap1 gene and promoting Nrf2 into the nucleus, then enhancing the expression of downstream HO-1 gene, thereby inhibiting the phosphorylation of STAT3, and then reducing the expression of RORγt (Fig. 6L). Therefore, *C. butyricum* may reduce the immune inflammatory response of PNS through the HO-1/STAT3/RORγt signaling pathway.

*C. butyricum* treatment improved the imbalance of the gut microbiome in mice with PNS.

**Fig. 7:**
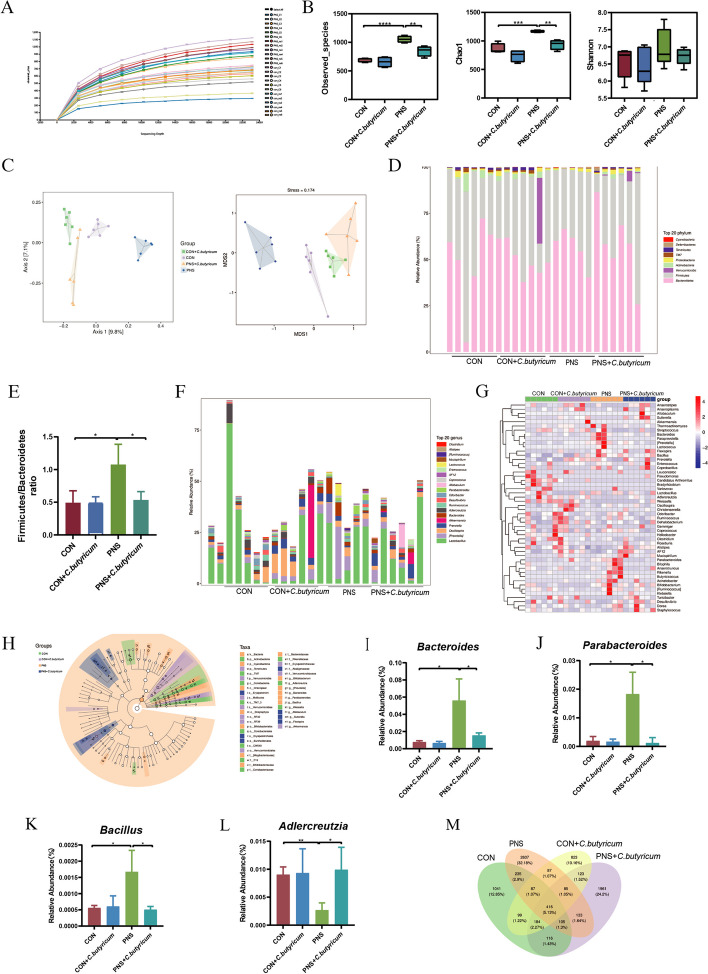
The modulation of gut microbiome by probiotic *C. butyricum* supplementation in mice with PNS. Dilution curve (**A**). Alpha diversity analysis included observed species, Chao1 and Shannon (**B**). Beta diversity analysis includes principal coordinate (PcoA) analysis and non-metric multidimensional scaling (NMDS) analysis (**C**). Relative abundance of microbial species at the phylum level (**D**). Ratio of *Firmicutes* to *Bacteroidetes* (**E**). Relative abundance of microbial species at the genus level (**F**). Heatmap of species composition at the genus level of species clustering (**G**). LEfSe analysis displaying of inter-group differential taxa based on taxonomic tree **H**. *Bacteroides* (**I**). *Parabcteroides* (**J**)**. **Bacillus (**K**). *Adlercreutzia* (**L**). Venn diagram **M**. Data were expressed as mean±SD. *P < 0.05, **P < 0.01, ***P < 0.001, ****P < 0.0001

In recent years, accumulating studies have confirmed that microbiota has an important impact on the occurrence and development of PNS through the gut-kidney axis [12, 17, 22]. Due to assess the impact of *C. butyricum* on the composition of gut microbiota in PNS, we detected fecal samples in different groups by 16S rRNA sequencing. Here, alpha diversity was first analyzed. The rarefaction curve showed that the sequencing depth of samples among different samples was consistent (Fig. 7A). Compared with the CON group, the observed species and Chao 1 of PNS mice were significantly increased (*P* < 0.0001, *P* < 0.001), while *C. butyricum* treatment significantly reduced the observed species characteristics and Chao 1 (all* P* < 0.01), but there was no significant difference in Shannon index among the groups (*P* > 0.05) (Fig. 7B). PCoA and NMDS were used to analyze the overall composition of the bacterial community a cluster represents a group. We found that the bacterial community between the PNS group and the CON group was significantly different and different clusters were formed after the supplementation of *C. butyricum* in PNS (Fig. 7C).

Next, we studied the abundance changes of intestinal flora in different groups at the phylum and genus levels. The results showed that at the phylum level, *Firmicutes* and *Bacteroidetes* were dominant in different groups. The proportion of *Firmicutes* or *Bacteroidetes* in the PNS group was separately higher than that in the CON group and the ratio of *Firmicutes* to *Bacteroidetes* was significantly increased (*P* < 0.05). However, in the PNS+*C. butyricum* group, the proportions of *Firmicutes* and *Bacteroidetes* in the gut microbial community were restored, and the ratio of *Firmicutes* to *Bacteroidetes* also decreased significantly (*P* < 0.05) (Fig. 7D and E). At the genus level, the top 20 genera were also analyzed and found differences among groups (Fig. 7F). The heatmap of the abundance data of the top 50 genera with average abundance showed that the abundance distribution in the PNS group was different from that in the other three groups (Fig. 7G). In addition, LEfSe analysis found that there were significant differences in species among groups (Fig. 7H). We observed that the abundances of *Bacteroides*, *Parabcteroides,* and *Bacillus* in the PNS group were elevated compared with those in the CON group (*P*<0.05), but the abundance of *Adlercreutzia* was lower in the PNS model (*P*<0.01). After dietary probiotic *C. butyricum* treatment, the abundances of *Bacteroides*, *Parabcteroides,* and *Bacillus* were reduced (*P*<0.05), whereas *Adlercreutzia* was increased (*P*<0.05) (Fig. 7I to L). Moreover, community analysis using the Venn diagram showed that the number of ASVs shared by the 4 groups was 416, and the number of ASVs unique to the CON group, CON+*C. butyricum* group, PNS group, and PNS+*C. butyricum* group was 1041, 823, 2607, and 1961, respectively (Fig. 7M). In summary, *C. butyricum* significantly changed the initial proportion of ASVs at the genus level, mainly including *Bacteroides, Parabcteroides, Bacillus,* and *Adlercreutzia.*

*C. butyricum* treatment modulated urinary microbiota in PNS mice.

**Fig. 8:**
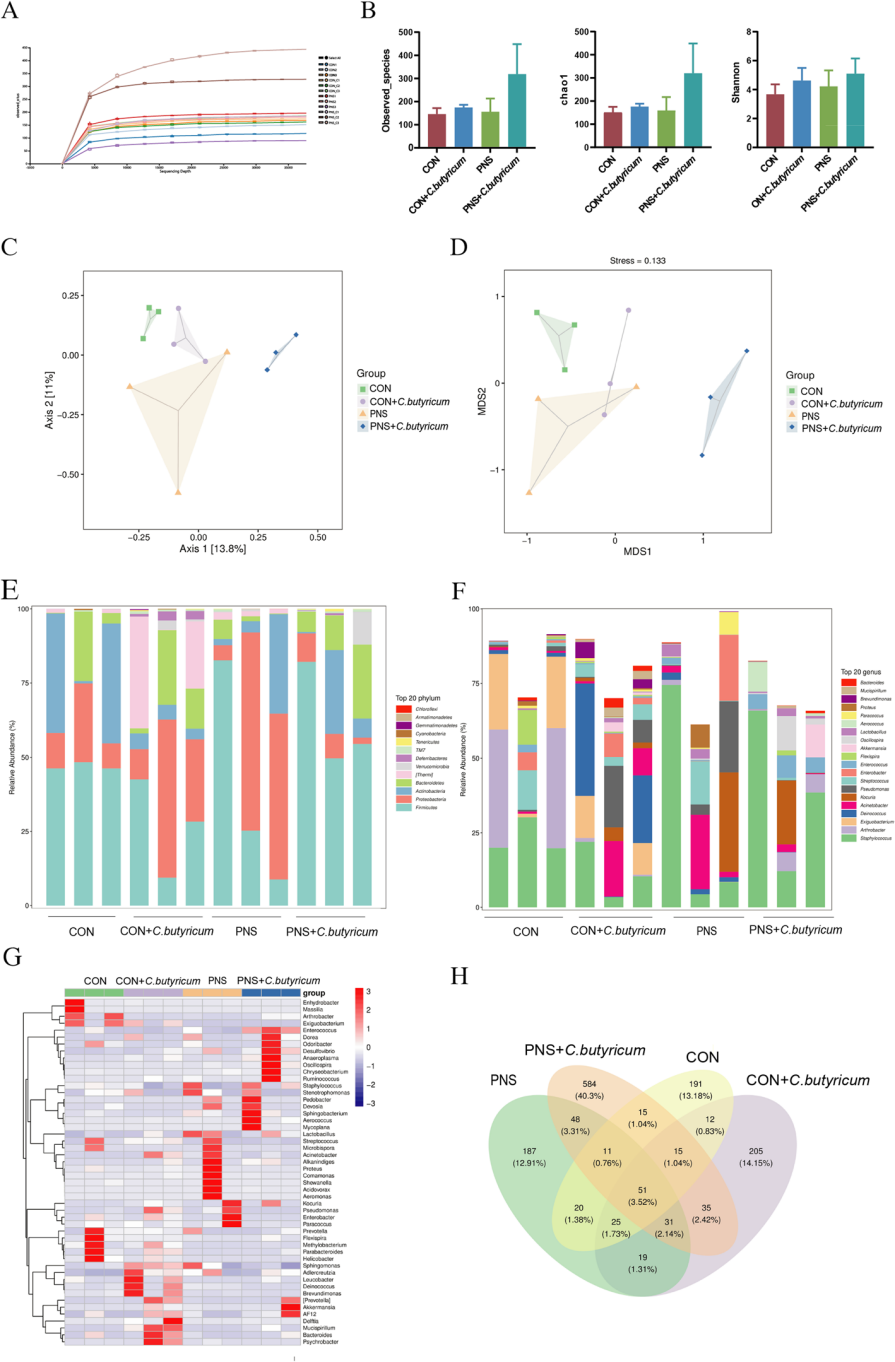
The changes of urinary microbiota after *C. butyricum* treatment in mice with PNS. Dilution curve (**A**). Alpha diversity analysis included observed species, Chao1 and Shannon (**B**). PcoA analysis (**C**). NMDS analysis (**D**). Relative abundance of microbial species at the phylum level (**E**). Relative abundance of microbial species at the genus level (**F**). Heatmap of species composition at the genus level of species clustering (**G**). Venn diagram (**H**)

Studies have shown that there are changes in the urinary microbiota in many urinary system diseases, such as interstitial cystitis, and bladder cancer [23, 24]. Thus, we speculated a close relationship between urinary microbiota and PNS with or without dietary *C. butyricum* treatment. After 16S rRNA sequencing and analysis of the urine microbiota in mice of 4 groups. The sparse curve showed that the sequencing depth among different samples was consistent, indicating that the amount of sequencing data was reasonable (Fig. 8A). There was no significant difference in alpha diversity between the PNS group and the CON group (Observation species, Chao1 index, shannon index) (Fig. 8B). PCoA and NMDS were used to analyze the overall composition of urinary microorganisms. PCoA results showed that mice in the CON group had different bacterial community composition from the PNS group. However, the total bacterial community was dramatically altered by *C. butyricum* treatment (Fig. 8C). NMDS analysis also obtained similar results (Fig. 8D).

At the phylum level, we found that *Firmicutes, Proteobacteria, Actinobacteria,* and *Bacteroidetes* were dominant. *Firmicute*s were more abundant in the CON, CON+*C. butyricum* and PNS+*C. butyricum* groups, but *Proteobacteria* was more abundant in the PNS group (Fig. 8E). At the genus level, we analyzed the top 20 genera of urinary microorganisms and found that there was no significant difference in species abundance between the groups (Fig. 8F). Further, the abundance data of the top 50 genera with average abundance were plotted to show that the species abundance distribution of the PNS group was different from that of other groups (Fig. 8G). In addition, the Venn diagram showed that there were 51 common microorganisms among diverse groups. There were 191 ASVs in the CON group, 205 ASVs in the CON+*C. butyricum* group, 187 ASVs in the PNS group, and 584 ASVs in the PNS+*C. butyricum* group (Fig. 8H).

*C. butyricum* treatment increased the contents of SCFAs in the feces of PNS mice.

**Fig. 9:**
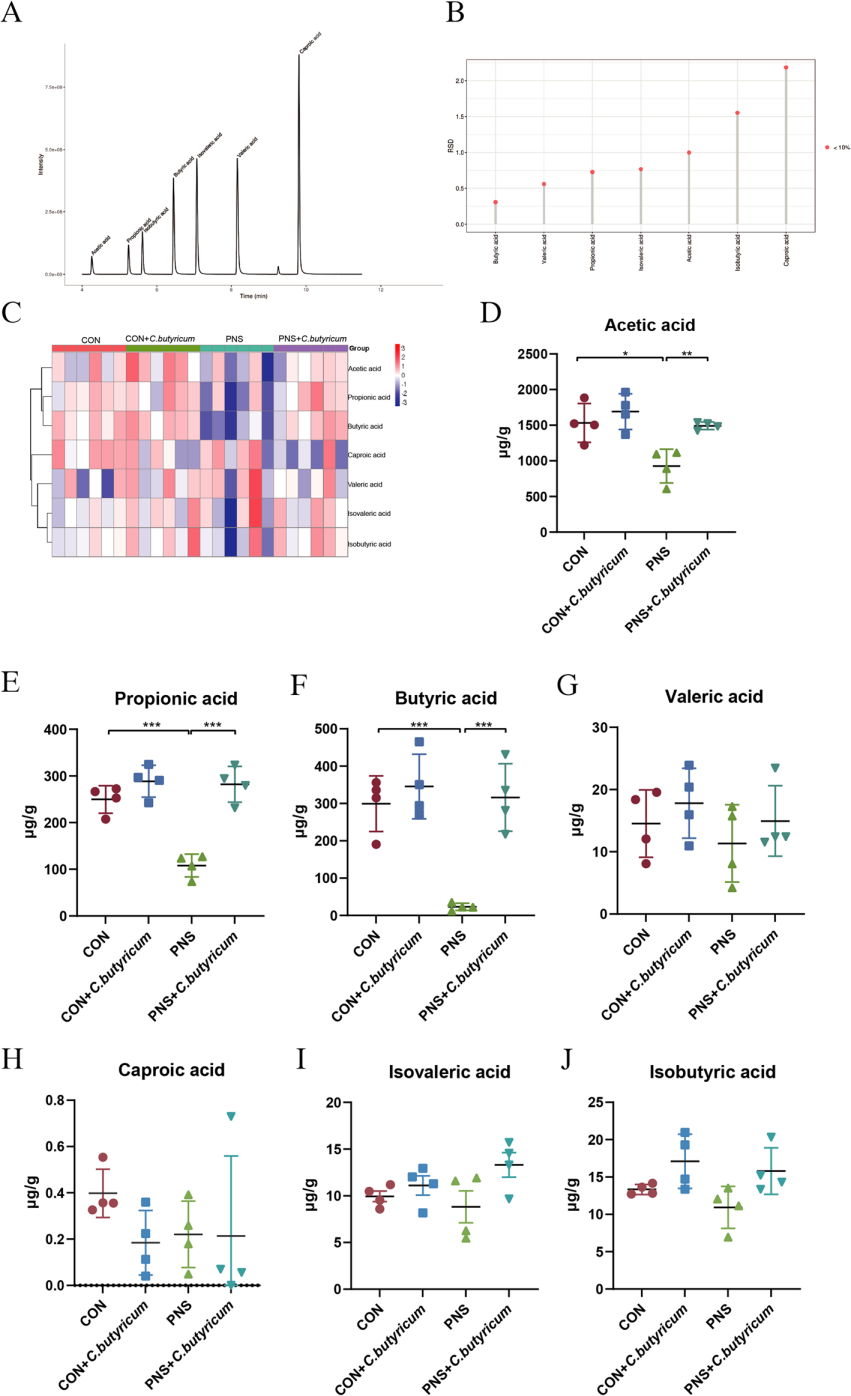
*C. butyricum* treatment increased the contents of short-chain fatty acids (SCFAs) in feces of PNS mice. Chromatogram of mouse fecal samples (**A**). Relative standard deviation (**B**). Cluster heatmap of whole metabolites of gut microbiota (**C**). Acetic acid (**D**). Propionic acid (**E**). Butyric acid (**F**). Valeric acid (**G**). Caproic acid (**H**). Isovaleric acid (**I**). Isobutyric acid (**J**). Data are expressed as mean±SD. *P < 0.05, **P < 0.01, ***P < 0.001

The concentrations of SCFAs in the feces of mice in each group were determined by GC-MS. The total ion current chromatogram (TIC) showed that the methodology was stable (Fig. 9A) and the relative standard deviation (RSD) was less than 10%, indicating that the data was reasonable (Fig. 9B). Then, the relative contents of SCFAs in each group were displayed using the overall metabolite clustering heatmap (Fig. 9C), suggesting a significant reduction of the contents of SCFAs including acetic acid (*P* < 0.05) (Fig. 9D), propionic acid (*P* < 0.001) (Fig. 9E), and butyric acid (*P* < 0.001) (Fig. 9F) in mice with PNS. These decreased SCFAs were remarkably restored by continuous dietary *C. butyricum* intervention. Meanwhile, there was no significant difference in the contents of valeric acid, caproic acid, isovaleric acid, and isobutyric acid among different groups (all *P* > 0.05) (Fig. 9G to J). The protective effect of *C. butyricum* on PNS may be due to the promotion of SCFAs production in the intestine.

The effect of *C. butyricum* treatment on the contents of SCFAs in the urine of PNS mice.

**Fig. 10:**
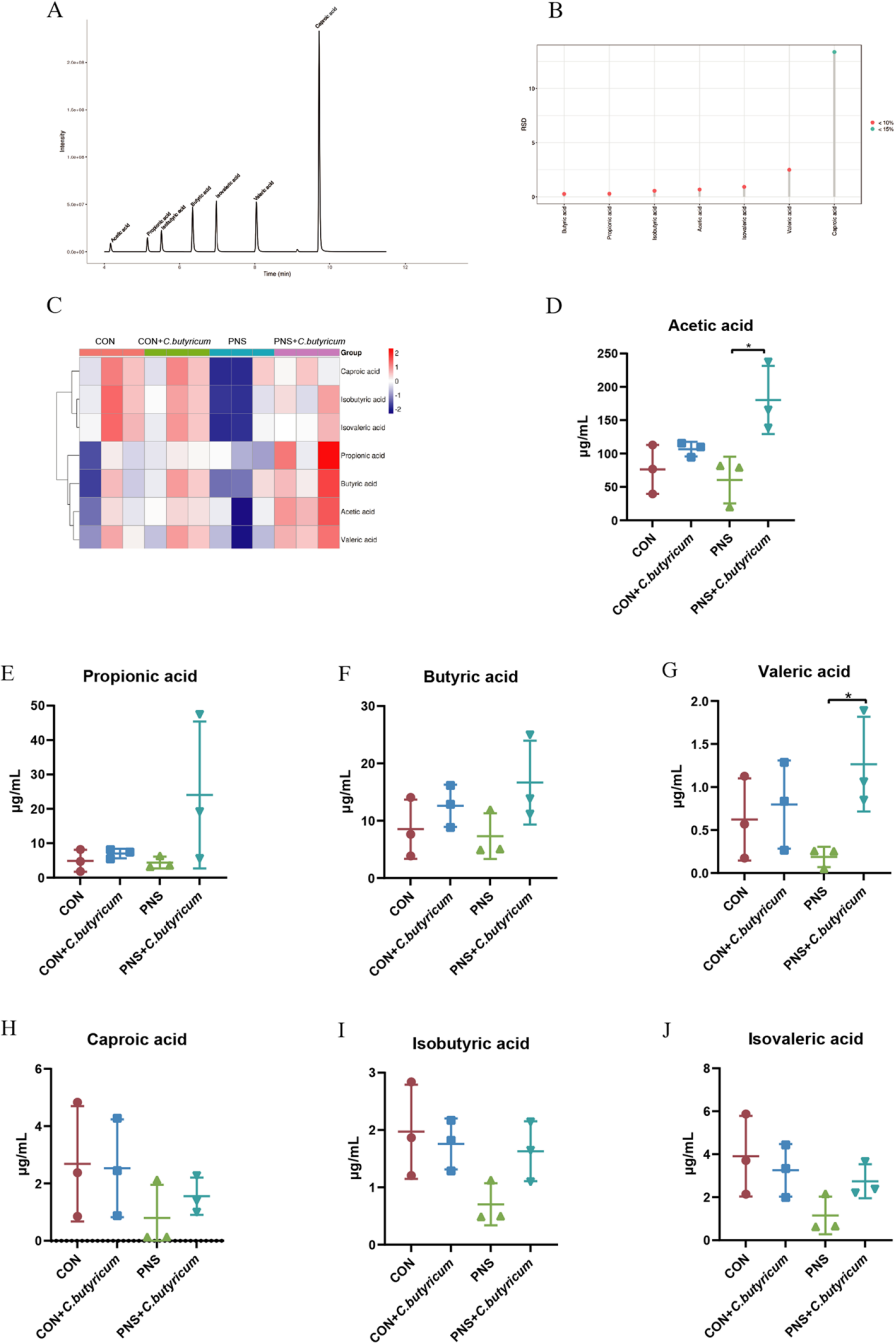
Effect of *C. butyricum* treatment on the contents of SCFAs in urine of PNS mice. Chromatogram of mouse urine samples (**A**). Relative standard deviation (**B**). Cluster heatmap of whole metabolites of urine microbiota (**C**). Acetic acid (**D**). Propionic acid (**E**). Butyric acid (**F**). Valeric acid (**G**). Caproic acid (**H**). Isobutyric acid (**I**). Isovaleric acid (**J**). Data were expressed as mean±SD. *P < 0.05

The above data that *C. butyricum* could promote the contents of SCFAs in the feces of PNS mice convinced us that the contents of SCFAs in the urine also may probably be influenced by dietary *C. butyricum* administration. Firstly, the method was proved to be stable according to TIC and the RSD was less than 10%/15%, indicating that the data performed well (Fig. 10A and B). The overall heatmap showed the relative contents of SCFAs in mice of diverse groups kept considerable difference (Fig. 10C). However, there was no significant difference in the contents of SCFAs between PNS group and CON group (*P* > 0.05) (Fig. 10D to J). Importantly, the treatment of *C. butyricum* increased the contents of acetic acid and valeric acid (all* P* < 0.05) (Fig. 10D and G). Therefore, dietary *C. butyricum* intervention significantly increased the content of acetic acid and valeric acid in urine.

### Correlation analysis

**Fig. 11:**
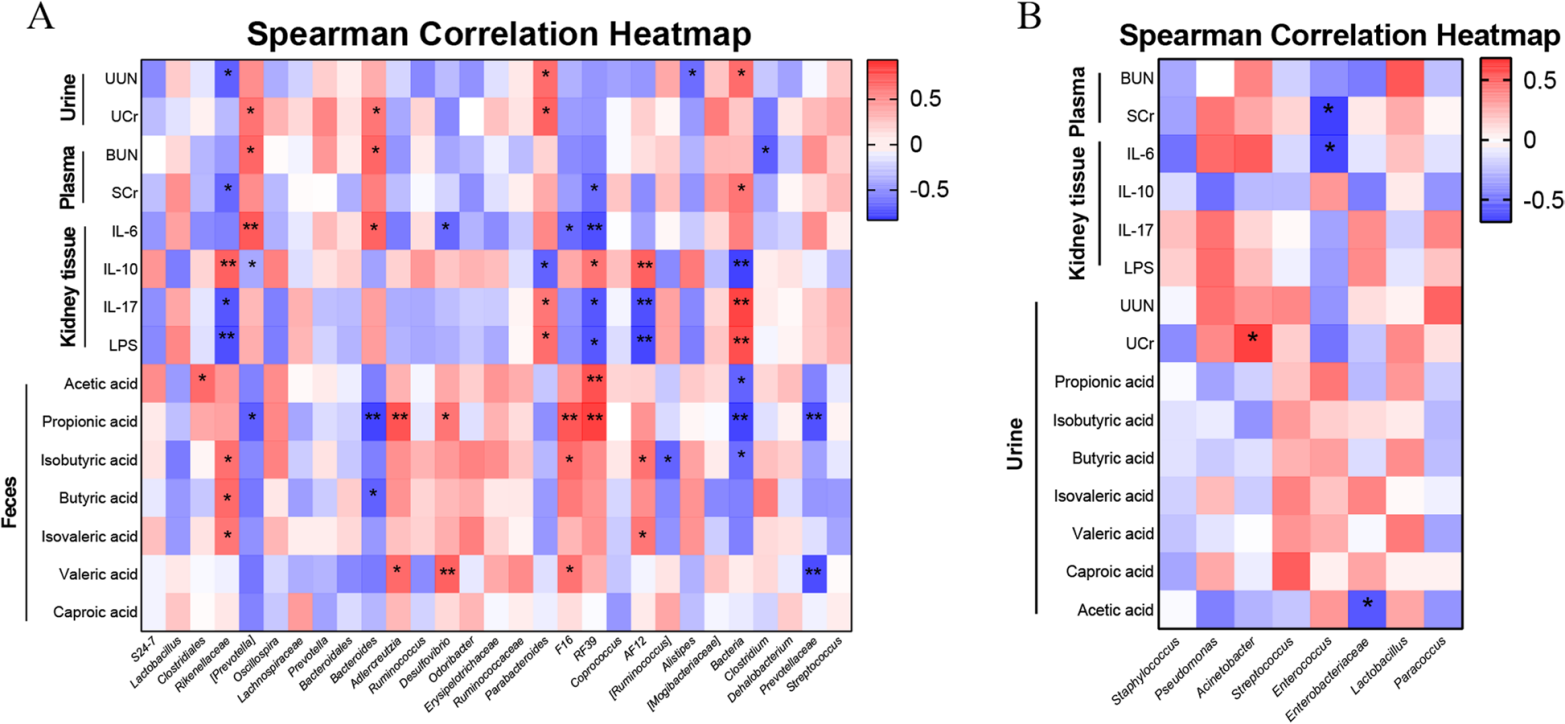
Correlation analyses among relative abundance of gut/urine microbiota and other related indicators. Correlation of gut microbiota with inflammation, metabolic indicators, and SCFAs in PNS (**A**). Correlation of urine microbiota with inflammation, metabolic indicators, and SCFAs in PNS (**B**). *P < 0.05, **P < 0.01

To evaluate the relationship among gut microbiome/urine microbiome, inflammation, metabolic indicators, and SCFAs in PNS, we performed a correlation analysis. We found in the gut microbial genus level that *Rikenellaceae, RF39,* and *AF12* were positively correlated with IL-10 and SCFAs. But it was inversely proportional to IL-6, IL-17, LPS, and metabolic indicators. On the contrary, *Prevotella, Bacteroides,* and *Bacteria* were negatively correlated with IL-10 and SCFAs positively correlated with IL-17, LPS, and metabolic indicators. The remaining bacteria including *Clostridiales, Adlercreutzia, Desulfovibrio,* and *F16* were positively correlated with SCFAs. In addition, *Parabacteroides* was directly proportional with urinary metabolism indicators, IL-17, and LPS*,* but inversely proportional to IL-10 (Fig. 11A). At the level of urinary microbial genera, only *Acinetobacte*r was correlated to UCr, *Enterococcus* was inversely proportional to SCr and IL-6, and *Enterobacteriaceae* was negatively associated with acetic acid (Fig. 11B). These results indicated that there was a closely complicated correlation among gut microbiome/urine microbiome, PNS metabolic indicators, and inflammatory indicators.

## Discussion

In this study, the protective effect of *C. butyricum* on PNS was investigated by measuring 24 h urinary protein, metabolites, kidney pathological damage, inflammation, and gut microbial community. Our results suggested that supplementation of *C. butyricum* could effectively alleviate PNS, which may be due to the regulation of Th17/Tregs balance by *C. butyricum* through HO-1/STAT3/RORγt signaling pathway to inhibit the immune inflammatory response of PNS and restore gut microbial disorders in mice. Herein, our study may provide a safe, effective, and inexpensive intervention for the treatment and prevention of the recurrence of PNS in children.

PNS in children is characterized by a series of pathological and physiological changes caused by a large amount of urinary protein loss [25]. The ultrastructure of podocytes is the ultimate barrier leading to the loss of urinary protein [2, 26]. Long-term proteinuria can lead to progressive decline in glomerular filtration function, kidney interstitial damage, kidney tubular sclerosis, and ultimately kidney dysfunction, development of chronic kidney disease, and poor prognosis [27]. In this study, it was observed that the BWs in mice with PNS induced by tail vein injection of DOX were lower, while *C. butyricum* treatment could slow down the weight loss of PNS. In addition, *C. butyricum* treatment reduced proteinuria in PNS, which was consistent with our conjecture. Importantly, compared to the PNS model, we found that after 6 weeks of intragastric administration of *C. butyricum*, urea nitrogen and creatinine in blood and urine, as well as pathological damage were alleviated, indicating that *C. butyricum* had a significant recovery effect on PNS injury.

At present, the pathogenesis of PNS is poorly understood. It is generally believed that immune abnormalities are the initial factors, and inflammatory response plays an important role in the occurrence and development of PNS [28, 29]. LPS is a highly inflammatory component of the cell wall of Gram-negative bacteria, which is the causal relationship between gut microbiota and systemic low-grade inflammation [30]. In this study, the levels of LPS in the gut and kidney were significantly reduced in the PNS+*C. butyricum* group, indicating that *C. butyricum* reduced the translocation and circulation of LPS from the intestine to the kidney in PNS, thereby helping to reduce the kidney inflammatory response, but the cellular and molecular mechanisms need further study. Tregs are a kind of significant immunosuppressive cells, represented by naturally generated thymus-derived CD4+CD25+Foxp3+ Tregs (nTregs) and inducible Tregs cells (iTregs) [31]. Under physiological conditions, Th17/Tregs are in a state of dynamic immune balance. When the body is abnormal, Th17/Tregs imbalance can cause a series of inflammatory immune responses to damage the body [9, 12, 32]. Studies have shown that Foxp3+ Treg cells in children with PNS are down-regulated, and the expression of IL-23p19, IL-17, IL-6, and IL-1β is increased [9]. Butyric acid can significantly enhancing the acetylation of histone h3 in the promoter of Foxp3 site and the conserved non-coding sequence region which is a key marker of Tregs, suggesting that butyric acid plays an important role in the induction of Treg cell differentiation [15, 18]. Our study also confirmed the downregulation of Treg cells and the up regulation of Th17 cells in PNS, importantly, both of which were restored after the intervention of *C. butyricum*. In addition, our study also observed that the anti-inflammatory factor IL-10 of PNS was significantly reduced and the pro-inflammatory factor IL-6 and IL-17A was significantly increased, which was significantly improved after *C. butyricum* intervention. In summary, *C. butyricum* can regulate the balance of Th17/Tregs to reduce the immune inflammatory response of PNS.

**Fig. 12:**
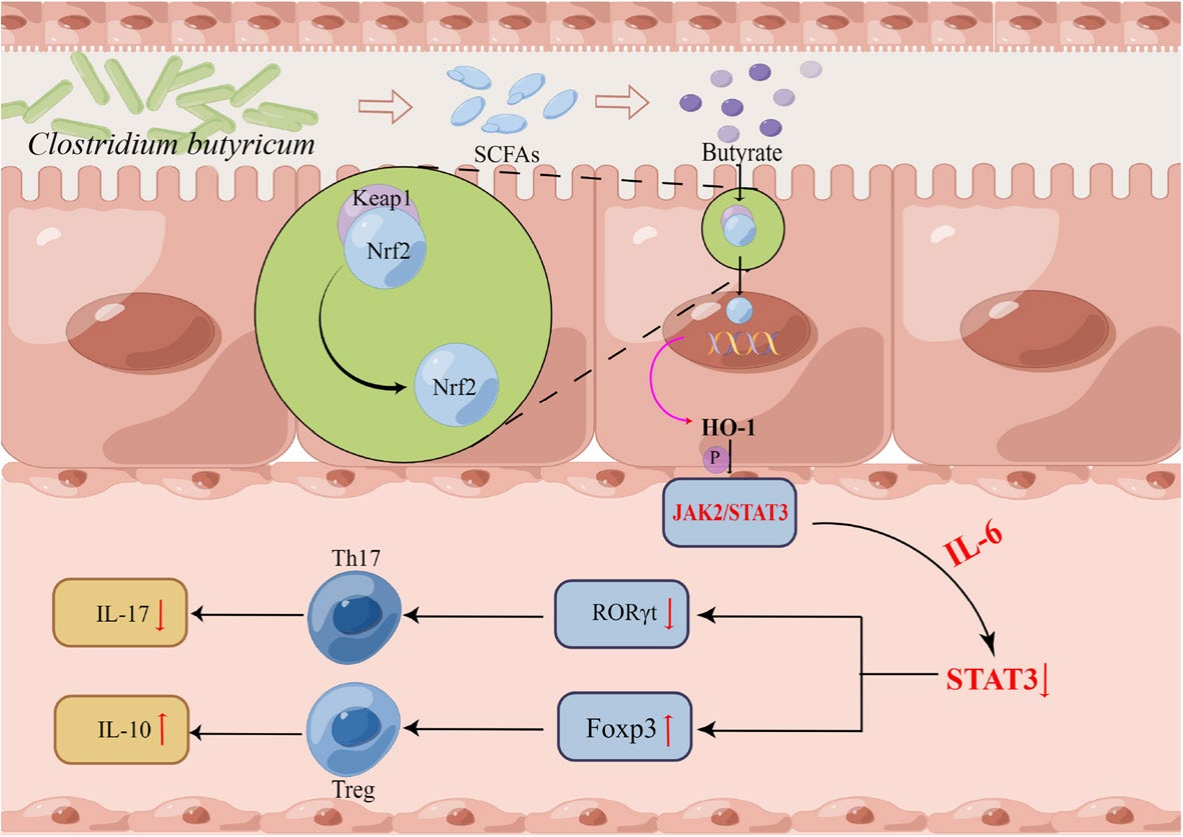
Patterns of effectiveness of *C. butyricum* for the treatment of PNS by regulating Th17/Tregs axis via the HO-1/STAT3/RORγt signaling pathway and modulating gut microbiota in mice

Studies have found that butyrate may activate Nrf2 at the transcriptional level, thereby triggering anti-inflammatory and antioxidant responses to prevent diabetes-induced body damage and slow down the damage of diabetic nephropathy [33]. Similar studies have demonstrated that butyrate blocks liver injury and cerebral ischemia injury caused by various factors by regulating the Keap1/Nrf2 pathway [34, 35]. Therefore, we speculate that *C. butyricum* may play a regulatory role in the Keap1/Nrf2 pathway through its metabolites. Further search for the downstream of Nrf2, in many tissues and organs of the organism, the antioxidant response element (ARE) maintains the redox state and reduces the oxidation pathway in oxidative stress under normal conditions. When the body is stimulated by oxidative stress, Nrf2 and Keap1 are separated from each other, enter the nucleus, and bind to ARE to activate the expression of antioxidant enzyme genes including Nicotinamide adenine dinucleotide phosphate (NADPH) and HO-1 [36–39]. More interestingly, HO-1 can inhibit the IL-6-induced STAT3 phosphorylation pathway. HO-1 promotes the formation of JAK2-STAT3 complex by binding to JAK2, inhibits the phosphorylation of STAT3, thereby down-regulating the expression of RORγt, and induces the differentiation of Treg cells by inhibiting Th17 cell differentiation [16, 40, 41]. In the present study, as we judged, the metabolite butyric acid of *C. butyricum* binds to keap1 to inhibit the expression of Keap1 gene, making the binding of Keap1 to Nrf2 unstable, Nrf2 detaches from Keap1 and binds to ARE, increasing the expression of downstream antioxidant enzyme HO-1 protein, thereby inhibiting the phosphorylation of STAT3, not JAK2, regulating the IL-6-STAT3-RORγt pathway to reduce the expression of RORγt, ultimately inhibiting the differentiation of initial CD4+ T cells into Th17 cells and promoting the converse differentiation into Treg cells (Fig. 12). In our study, the decrease of HO-1 protein expression level in PNS mice was in line with expectations, while the gene expression level of HO-1 was increased. In summary, dietary *C. butyricum* supplementation reduced the immune inflammatory response of PNS through the HO-1/STAT3/RORγt signaling pathway.

The importance of intestinal microbes in nephrotic syndrome has been widely recognized [12, 42, 43]. For gut microbiota, we found that *Firmicutes* and *Bacteroidetes* were dominant in different groups, which was consistent with previous studies [22]. Moreover, the increase in the ratio of *Firmicutes*/*Bacteroidetes* in the PNS group was a feature identified with gut dysbiosis, which was closely related to kidney injury [44]. At the genus level, our results showed that *C. butyricum* restored intestinal homeostasis of PNS by up-regulating *Adlercreutzia* and down-regulating *Bacteroides*, *Parabacteroides*, and *Bacillus.* Studies have shown that *Adlercreutzia* is depleted in patients with liver diseases such as NAFLD, and its abundance also decreases with the progression of the disease, indicating a close correlation with the severity of the disease [45]. In this study, *Adlercreutzia* was positively correlated with IL-10 and SCFAs, but it is inversely proportional to IL-17, LPS, and PNS metabolic indicators, indicating that *Adlercreutzia* may contribute to the anti-inflammation effect on PNS in mice.

SCFAs are the main metabolites produced by bacterial fermentation of dietary fiber in the gastrointestinal tract [46]. Numerous evidences have suggested that SCFAs play a key role in regulating mental function [47], metabolism [48, 49], inflammatory response [50], and other diseases. The highest contents of SCFA in the intestine were acetate, propionate, and butyrate. Acetic acid was mainly produced by anaerobic bacteria, such as *A.muciniphila* and *Bacteroides* spp. Propionate was mainly produced by *Bacteroides,* and Butyrate was mainly produced by *Clostridium* cluster IV and XIVa, and *F.prausnitzii * [46]. A study has shown that butyric acid is less in the feces of chronic kidney disease (CKD) mice and *prausnitzii* could increase the level of butyric acid in feces. No effect of *prausnitzii* on acetic acid or propionic acid was found [45]. More interestingly, in our study, it was found that *Bacteroides* was negatively correlated with SCFAs. The abundance of *Bacteroides* in PNS group was significantly increased, while the contents of acetic acid, propionic acid, and butyric acid were significantly decreased. The contents of acetic acid, propionic acid, and butyric acid were significantly increased after *C. butyricum* treatment. Additionally, there may be other microbial metabolites in the role of PNS to be further studied using metabolomics methods.

Studies have shown that urinary microbes play an important role in the development of urolithiasis, interstitial cystitis, bladder cancer, and attention deficit hyperactivity disorder (ADHD) [24, 24, 51–53]. However, the role of urethral microbes in the development of PNS has rarely been reported. In this study, we found that there was no significant difference in alpha diversity between PNS group and the CON group, and beta diversity showed different bacterial community composition. At the phylum level, we found that *Firmicutes*, *Proteobacteria*, *Actinobacteria*, and *Bacteroidetes* were dominant, and *Proteobacteria* was more abundant in the PNS group. *Proteobacteria* is a human opportunistic pathogen that mainly causes infections in people with impaired immune systems and causes complex urinary tract infections [54]. Whereas, in the genus level, we found no significant difference in species abundance between groups. Our study also showed that there was no statistically significant difference in the content of short-chain fatty acids in PNS urine, but *C. butyricum* treatment increased the content of acetic acid and valeric acid in urine. Indeed, the exact role of urinary microorganisms in PNS needs further study.

## Conclusion

In summary, our current study demonstrates that the probiotic *C. butyricum* ameliorates PNS inflammation by regulating Th17/Tregs balance through the HO-1/STAT3/RORγt signaling pathway and reshaping gut/urine microbiota in the gut-kidney axis, which may be a safe and inexpensive treatment for PNS.

### Supplementary Information

**Supplementary Material 1.**

## Data Availability

The datasets presented in this study can be found in online repositories. Accession number(s): NCBI, PRJNA10285250.

## Abbreviations

PNS: Primary nephrotic syndrome
*C. butyricum*: *Clostridium butyricum*
DOX: Doxorubicin hydrochloride
BUN: Blood urea nitrogen
SCr: Serum creatinine
UUN: Urine urea nitrogen
UCr: Urine creatinine
LPS: Lipopolysaccharides
Nrf2: Nuclear factor erythroid2-related factor 2
Keap1: Kelch-like ECH-associated protein 1
HO-1: Heme oxygenase-1
STAT3: Signal transducer and activator of transcription 3
JAK2: Janus kinase 2
RORγt: Retinoic acid-related orphan receptor gamma t
SCFAs: Short-chain fatty acids
