# Supplementary figures and images for: Clostridium butyricum inhibits the inflammation in children with primary nephrotic syndrome by regulating Th17/Tregs balance via gut-kidney axis
Source: BMC Microbiol. 2024 Mar 23;24:97. doi: 10.1186/s12866-024-03242-3 (PMC10960420; doi:10.1186/s12866-024-03242-3)

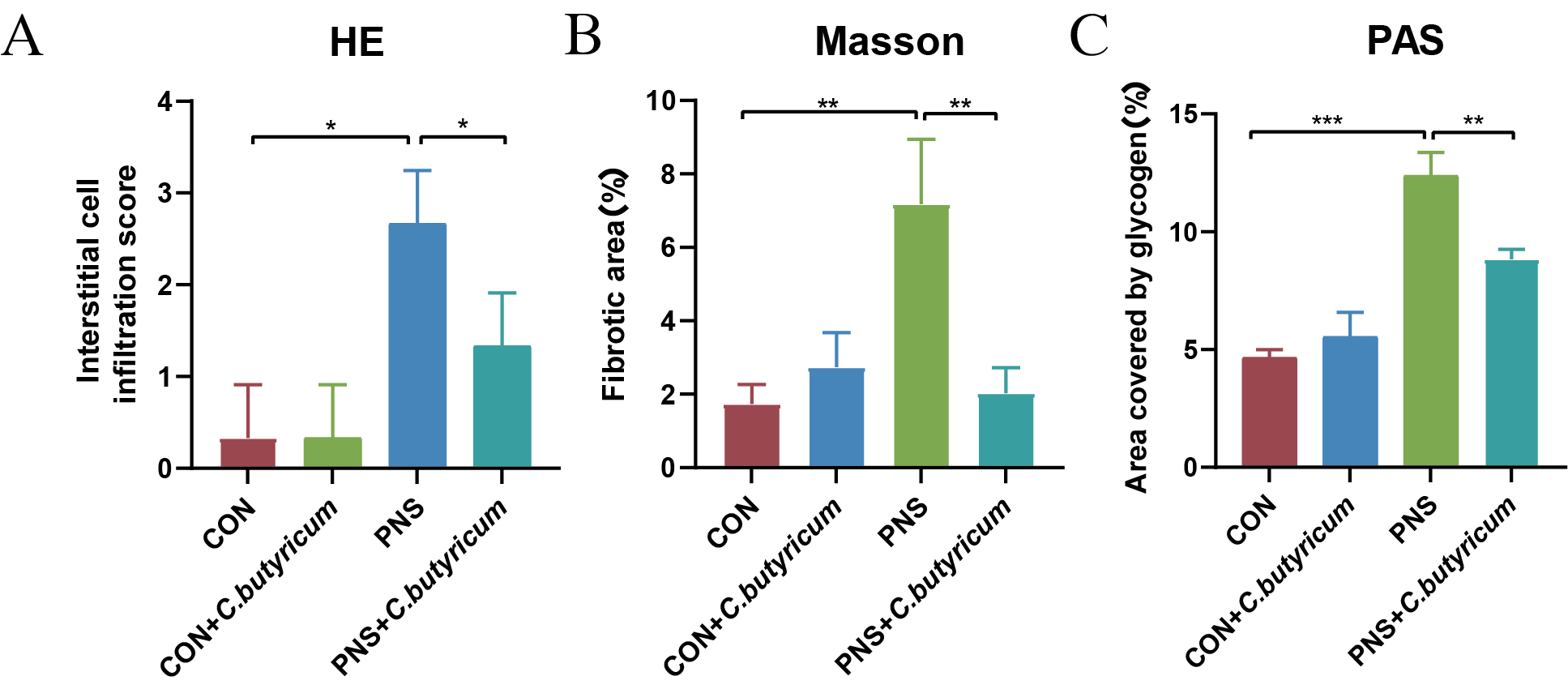

Supplement: Supplementary file 1 — Supplementary Material 1. [file 12866_2024_3242_MOESM1_ESM.tif]
